# Metagenome Sequences of a Wastewater Treatment Plant Digester Sludge-Derived Enrichment Culture

**DOI:** 10.1128/MRA.00712-20

**Published:** 2020-08-06

**Authors:** Heiko Nacke, Laura L. Kirck, Sophia Schwarz, Dominik Schneider, Anja Poehlein, Rolf Daniel

**Affiliations:** a Genomic and Applied Microbiology and Göttingen Genomics Laboratory, Institute of Microbiology and Genetics, Georg-August University of Göttingen, Göttingen, Germany; University of Southern California

## Abstract

We sequenced the metagenome of a microbial community enriched under strictly anaerobic conditions from wastewater treatment plant-derived digester sludge. The metagenomic analysis of the enrichment revealed that *Acetobacterium* and methanogenic archaea belonged to the dominant prokaryotes, and genes encoding components of the Wood-Ljungdahl pathway were identified.

## ANNOUNCEMENT

Anaerobic fermentation is performed in digestion tanks of wastewater treatment plants. During this process, biogas usable for energy production is released. A major fraction of biogas release results from methanogenesis conducted by various archaea during anaerobic digestion (1). These microorganisms utilize H_2_, CO_2_, and acetate, which is produced by acetogens (2). Acetogenic bacteria are increasingly recognized by industry, as CO_2_/H_2_ and syngas are interesting new substrates for biotechnological processes (3). In this study, we enriched acetogens thriving in digester sludge and performed metagenomic analysis.

In the first step, sludge was collected from a digestion tank of the wastewater treatment plant in Göttingen, Germany (51°33′11.0ʺN, 9°55′07.2ʺE). Subsequently, 50 ml DSMZ 311 medium (Deutsche Sammlung für Mikroorganismen und Zellkulturen [DSMZ], Braunschweig, Germany) was inoculated with 1 ml collected sludge in a 250-ml Afnor plasma bottle sealed with a butyl rubber plug. An incubation at 30°C for 5 days under anaerobic conditions and an H_2_/CO_2_ atmosphere (H_2_, 66% [vol/vol]; CO_2_, 33% [vol/vol]) followed. Metagenomic DNA was isolated from the digester sludge-derived enrichment culture using the MasterPure complete DNA purification kit according to the manufacturer’s protocol (Epicentre, Madison, WI, USA). The purified DNA was used to generate paired-end sequencing libraries with the Nextera DNA sample preparation kit (Illumina, San Diego, CA, USA), which were sequenced by employing the MiSeq reagent kit version 3 and a MiSeq instrument as recommended by the manufacturer (Illumina). The resulting data of two sequencing runs based on the same sequencing library were pooled prior to further processing. All of the following processing steps were based on the pooled data. Default parameters were used for all software unless otherwise specified. Quality trimming of reads was performed by applying the program fastp 0.19.6 (4) and yielded 18,461,148 paired-end reads. Average read lengths of 173 and 168 bp were recorded for the forward reads and the reverse reads, respectively. Based on fastp quality filtering, sequences shorter than 50 bp were removed, and base correction in overlapping regions of each pair of reads was performed. In addition, 5′ and 3′ end trimming with a sliding window of 4 was performed. Furthermore, the adapter sequence autodetection for paired-end data was enabled, and the parameter “qualified_quality_phred” was set to 20. For taxonomic classification of trimmed sequences, Kaiju 1.6.3 (5), with the NCBI nonredundant protein database 2018-11-04 as reference, was used. In the next step, all reads were *de novo* assembled with metaSPAdes 3.13.0 (6), resulting in 77.52% of all reads assembling into 9,153 contigs of ≥2,000 bp. Subsequently, annotation was performed by employing Prokka 1.14.0 (7) in meta mode to improve gene predictions for highly fragmented genomes.

Approximately 57 and 43% of the quality-controlled reads were classified as derived from bacteria or archaea, respectively. The latter prokaryotic group mainly comprised the genera *Methanoculleus* (17%) and *Methanospirillum* (8%). Bacteria were dominated by the phyla *Firmicutes* (22%) and *Bacteroidetes* (22%). Within the phylum *Firmicutes*, *Clostridia* (17%) and *Acetobacterium* (11%) represented the most abundant class and genus, respectively, whereas *Bacteroidetes* were dominated by *Porphyromonadaceae* (11%). Analysis of Prokka-based annotation with respect to the assembled contigs revealed the presence of several Wood-Ljungdahl pathway genes. These genes include, e.g., *ascA*, *ascC*, *fhs*, *folD*, and *gcvH*. Furthermore, genes coding for key enzymes of butanol fermentation, such as butyryl-coenzyme A (CoA) dehydrogenase (*bcd*), 3-hydroxybutyryl-CoA dehydrogenase (*hbd*), and 3-hydroxybutyryl-CoA dehydratase (*crt*), as well as genes encoding the Rnf complex were detected in the metagenome.

### Data availability.

This whole-genome shotgun metagenome project, including the assembly, has been deposited at GenBank under the accession number JABNAD000000000. The version described here is version JABNAD010000000. The raw reads resulting from two Illumina MiSeq runs were submitted to the NCBI Sequence Read Archive (SRA) under the accession numbers SRR8846821 and SRR8846822. Both corresponding sequencing runs were based on the same sequencing library and pooled prior to assembly.
